# Impact of Bi_2_O_3_ on prepared nano (SiO_2_-Na_2_O-CaO-B_2_O_3_) glass as radiation shielding material

**DOI:** 10.1038/s41598-024-67363-5

**Published:** 2024-08-03

**Authors:** A. S. Doma, Mahmoud I. Abbas, Abd El Hady B. Kashyout, Ebrahim A. Mahdy, Eman A. Ghafeir, Mirvat Fawzi Dib, Hala Abdellatif, Ahmed M. El-Khatib

**Affiliations:** 1 Advanced Technology and New Materials Research Institute (ATNMRI), City of Scientific Research and Technological Applications (SRTA-City), New Borg El-Arab City, 21934, Alexandria, Egypt; 2 Physics Dept., Faculty of Science, Alexandria University, Alexandria, 21321 Egypt; 3 Glass Research Department, National Research Centre, El-Buhouth St, Dokki, Cairo, 12622 Egypt; 4 Clinical Oncology and Nuclear Medicine Department, Faculty of Medicine, Alexandria University, Alexandria, 21511 Egypt

**Keywords:** Materials science, Nanoscience and technology, Physics

## Abstract

Melt quenching technique was used to create Bismuth Boro-Silicate nano glasses with compositions of 45SiO_2_-10CaO- 25Na_2_O- xBi_2_O_3_- (20-x) B_2_O_3_ (where x is 0, 5, 10, 15, and 20 mol %). Standard point sources AM-241, Ba-133, Co-60, Cs-137, and Eu-152 were used in the radiation experiment to evaluate the attenuation coefficients spanning the energy range of 59.51 keV to 1048.01 keV. The findings show that adding Bi_2_O_3_ in place of B_2_O_3_ increases the following: radiation protection efficiency (RPE%), transmission factor (TF%), absorption buildup factor values (ABF), exposure buildup factor values (EBF), mass attenuation coefficients (MACs), linear attenuation coefficients (LACs), and radiation protection efficiency (RPE%). In comparison to lead glass, these findings demonstrate the potential of nano Bismuth Boro-Silicate glass as a radiation shielding material.

## Introduction

There are many different kinds of radiation, mostly charged and uncharged. Charged types, including neutrons and gamma rays, interact very strongly and produce different charged particles through absorption mechanisms^1^. In addition to their harmful effects, gamma rays are used in many different fields; therefore, when using them, one should also be shielded against their harmful effects. Exposure to γ-rays for extended periods of time can also cause harmful effects on human cells. As a result, a lot of researchers concentrated on talking about radiation shielding materials^2,3^. Due to its high density and consequent effectiveness, traditional shielding materials like lead or lead-based compounds are utilized in shielding. However, lead has many drawbacks as well, including being hazardous to people and the environment^4^.When alpha or beta particles decay, atomic nuclei release gamma rays, which are extremely powerful electromagnetic waves^5,6^. The offspring nucleus may be in an excited (unstable) state and have extra energy when the original nucleus breaks apart and releases an alpha or beta particle^7,8^. Glass and glass-based materials have been investigated as alternatives to lead because of glass’s amorphous nature and capacity to transmit light through its medium. Glass is therefore a crucial component for a wide range of applications worldwide because of its optical transparency. As an alternative to lead, bismuth (Bi) has a significant function in radiation shielding due to its density and high gamma attenuation coefficient. The aforementioned substitute, bismuth (Bi), has been researched the most and is presently a significant player in radiation shielding. Bismuth enhances chemical resistance and aids in stabilizing the glass structure. Furthermore, bismuth boosts the glass’s ability to guard against gamma rays because of its high atomic number^9^. Glass has become more widely used in a variety of technical applications over time. Boro-Silicate glasses have garnered significant attention, and recent research has examined silicate, Boro-Silicate, and other glass types in the context of radiation shielding^10–12^.Because of the requirement for materials that are transparent, inexpensive, chemically, mechanically, and thermally robust, as well as having a high level of resistance to radiation and absorption, glass alternatives have been created with regard to the shielding function. The primary factor driving glass shielding’s popularity in radiation protection technology is its easily mutable chemical structure, which can result in a wide range of combinations of physical, chemical, and radiation shielding qualities^13–15^.The performance of different glazing systems for radiation shielding has already been reported by the authors, both experimentally and theoretically^16–22^. A low-cost and efficient method for creating a uniform dispersion of Gd nanocomposite sheeting from surface-modified nanoparticles is presented by Ly B.T. La et al. With varying ϕs of 0.10, 0.12, and 0.14, a homogeneous 8 mm thick Gd2O3/epoxy nanocomposite can offer radiation protection efficiency equivalent to 0.25, 0.35, and 0.5 mm Pb equivalents. At higher energies, it can even surpass the corresponding Pb equivalents^23^. Kilic G. et al.^24^ synthesized a set of glasses with the nominal composition of xTeO2. (25-y)B2O3. ZV2O5.yYb2O3.They were therefore characterized in terms of their optical, structural, physical, thermal, and radiation attenuation properties. In order to achieve this goal, a wide range of analyses were conducted using both modeling and experimental methodologies. The synthesized glasses’ FTIR band locations and Gaussian band areas were examined. The shielding properties against nuclear radiation were assessed. Since the TBVY1.5 with the highest Ytterbium (III) oxide additive had excellent gamma-ray attenuation properties, it was evident that Ytterbium (III) oxide reinforcement had a surprising favorable impact on nuclear radiation attenuation properties. According to the results of a recent study, glasses reinforced with ytterbium (III) oxide and tellurium oxide may be a good candidate material for nuclear shielding.

Using the standard melt quenching method, glasses of 20 PbO–20 CaO–20 Sand–(40-x) B_2_O_3_–xCeO_2_ were created, with x = 0 (BCe-00), 2.5 (BCe-2.5), 5 (BCe-5.0), 7.5 (BCe-7.5), and 10 (BCe-10) in weight percentage^25^. An examination of the produced glasses using Fourier transform infrared (FTIR) spectroscopy has been carried out. We have looked at how the concentration of cerium (Ce) affects the suggested glasses’ ability to shield photons. These studies have also demonstrated the possibility for producing B_2_O_3_-based glasses for use as radiation shielding and their importance in a number of technologies. The optical qualities and radiation shielding of glass passed over boric oxide can only be improved by adding a number of components^25^. Utilising a narrow beam transmission method, our study aims to compare the radiation shielding properties of Bi2O3-incorporated Boro-Silicate glass with lead glass used in the radiation protection domain at 59.51, 80.99, 121.78, 244.7, 356.01, 661.65, 778.9, 964.08, 1173.24, 1332.5, and 1408.01 keV photon energies ^26^. This article’s goals were to produce new Boro-Silicate glass containing Bi2O3 and assess its radiation shielding capabilities. The melt-quenching approach was then used to manufacture glasses with the composition of Bi2O3, Na2O, CaO, SiO2, and B2O3^27,28^.

## Material and measurement

### Glass synthesis

**Figure 1:**
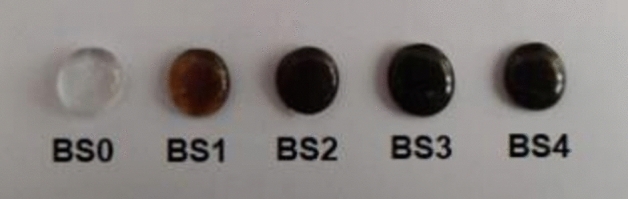
The investigated glass samples.

In this work, a typical melt quenching procedure was used to synthesise a series of glass samples based on the 25Na_2_O-10CaO- (20-x) B_2_O_3_- xBi_2_O_3_-45SiO_2_ (mole %) system, where X = 5, 10, 15 and 20 mol %). The glasses were then classified into 5 samples, with composition displayed in Table 1. The glass batch compositions were made using high purity grade reagents (purity > 99.9%) of silica (SiO_2_), boric acid (H_3_BO_3_), bismuth oxide (Bi_2_O_3_), calcium carbonate (CaCO_3_), and sodium carbonate (Na_2_CO_3_). In an agate mortar, a 30 g batch of each composition was thoroughly mixed. The batches were then melted in an electrical oven (Carbolite HTC 1550 ºC, Vechstar Sheffield, United Kingdom) for one hour at a temperature between 900 and 1000 °C. Subsequently, the glass is melted at room temperature and cast into stainless-steel moulds that have been prepared to create the desired forms for the measurements. After cooling to below 50 °C, or the glass transition temperature, Tg (Tg-50), the quenched glass species were placed in an annealing furnace and left there for 30 min. Subsequently, the furnace is turned off, and the glass specimens gradually cool down at room temperature to relieve any residual stress. This produces stiff glass samples that are free from tension and strain, and they also stabilise. The created samples were then divided into pieces with a thickness of approximately 0.55 cm, as illustrated in Fig. 1.

**Table 1:** The oxide constituents of the studied glass compositions.

Sample codes	Oxide constitutions (mole %)				
	Na_2_O	CaO	Bi_2_O_3_	B_2_O_3_	SiO_2_
BS0	25	10	0	20	45
BS1	25	10	5	15	45
BS2	25	10	10	10	45
BS3	25	10	15	5	45
BS4	25	10	20	0	45

### Measurement of radiation shielding properties

**Figure 2:**
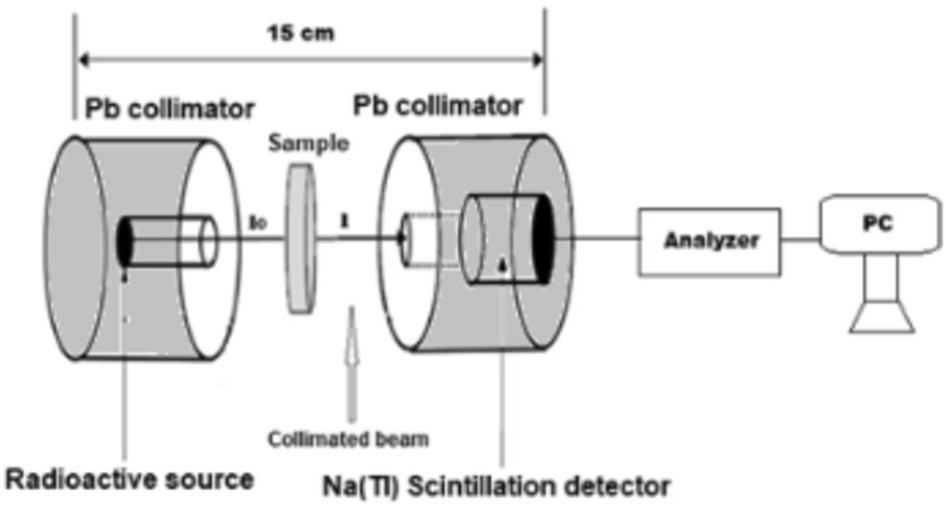
The narrow beam transmission geometrical setup.

The interaction between gamma rays is described by Lambert’s law in accordance with the photon flux of gamma rays through a material. To determine the transmitted gamma rays, use formula (1).

1 $$ {\text{I }} = {\text{ I}}_{0} {\text{e}}^{ - \mu t} $$

where μ is the linear attenuation coefficient, t is the glass sample’s thickness, I is the attenuation beam intensity, and I_0_ is the incident beam intensity. μ depends on the input photon’s energy, the medium’s density, and the element’s atomic number^26^. Figure 2 depicts the regulation that was applied during the experiment.

The XCOM program provides fundamental parameters that illustrate how a shielding material responds to radiation. These include the linear attenuation coefficient, or LAC or (μ), and the mass attenuation coefficient, or MAC or (μm). These parameters are essential for perceiving the physical shielding characteristics of materials. The total mass attenuation coefficient, or μm, of a given chemical or element mixture can be calculated using the formula in Eq. (2) [3, 5, 6,]

2 $${\mu }_{m}={\Sigma }_{i}\left({w}_{i}\times {\left(\frac{\mu }{\rho }\right)}_{i}\right)$$

while as ${w}_{i}$ is the weight fraction and ${\left(\frac{\mu }{\rho }\right)}_{i}$ *i* is the mass attenuation (${\mu }_{m}$*)* of the i_th_ element and $\mu$ is the linear attenuation coefficient.

3 $${\mu }_{m}=\frac{\mu }{\rho }$$

(${\mu }_{m}$ is measured in cm^2^/g).

### Calculation of gamma-ray buildup factor

Similar in significance to the atomic number of an individual element, the corresponding atomic numeral Zeq is a parameter; however, unlike atomic number, Zeq is dependent on photon energy. The equivalent atomic number, Zeq, for a given material was determined by using the WinXCom computer program (originally developed as XCOM). This involved comparing the ratio of MAC Compton to MAC Total of the glass samples at a given energy with the corresponding rate of an element at the same energy. As a result, for the elements and selecting materials in the energy range of 0.015–15 MeV, the Compton fractional mass attenuation coefficient, MAC Compton, and the total mass attenuation coefficients, MAC Total, have been determined^30,31^. The following formula might be computed in order to evaluate Zeq as follows for the fulfillment of Zeq at which the attribution value of MAC Compton / MAC Total was between two successive ratios of elements.

4 $$ Zeq = \frac{{Z1\left( {\log R2 - \log R1} \right) + Z2\left( {\log R2 - \log R1} \right)}}{\log R2 - \log R1} $$

where the atomic values Z1 and Z2 correspond to the corresponding ratios R1 and R2, respectively. R is a given glass’s ratio at a certain energy. The evaluated Zeq values of the glass samples are then used to examine the G-P fitting parameters (b, c, a, XK, and d) using the following equation^30,31^.

5 $$P=\frac{P1\left(\text{log}Z2-\text{log}Zeq\right)+P2(\text{logZeq}-\text{logZ}1)}{\text{log}Z2-\text{logZ}1}$$

The atomic numbers Z1 and Z2 are represented by the G-P fitting parameters P1 and P2, respectively. The accumulation factor data for 23 elements, one compound, and two admixtures at energies in the range of 0.015–15 MeV up to penetration depths of 40 mean free paths in utilizing the G-P approach were recently presented in American National Standards ANSI/ANS 6.4.3^29^. Therefore, the ABF (Absorption build up factor) and EBF (Exposure build up factor) of glass batches are used to determine gamma ray buildup factors^30,31^.

6 $$K\left(E,X\right)=c{x}^{a}+d\frac{\text{tanh}( \frac{x}{{X}_{k}} - 2 ) -\text{ tanh}(-2)}{1-\text{ tanh}(-2)} \text{for x }\le 40\text{ mfp}$$

where a, c, d, and Xk show the G-P fitting parameters, E is the photon’s energy, and x represents the depth of penetration into the mfp. The variation in photon dose and the shift in the spectrum’s format are represented by the parameter K.

#### Electron microscope for transmission (TEM)

High-resolution pictures of materials are obtained using TEM for chemical and structural characterisation. Glass samples were size, shape, and chemical composition assessed using a TEM in a JEOL-JEM 2100 F microscope. Throughout this process, sample containers made of copper grids were used.

#### Fourier transform infrared attenuated total reflectance (ATR-FTIR)

ATR is a sampling strategy that is connected to infrared spectroscopy. It is a useful technique for identifying the functional groups in materials that are difficult to analyse using conventional sample procedures, such as rubber and resins. The generated composites were subjected to ATR-FTIR measurements utilising a Platinum ATR device connected to a Bruker VERTEX 70 spectrometer running in the 4000–400 cm^–1^ range.

## Results and Discussion

### TEM images

**Figure 3:**
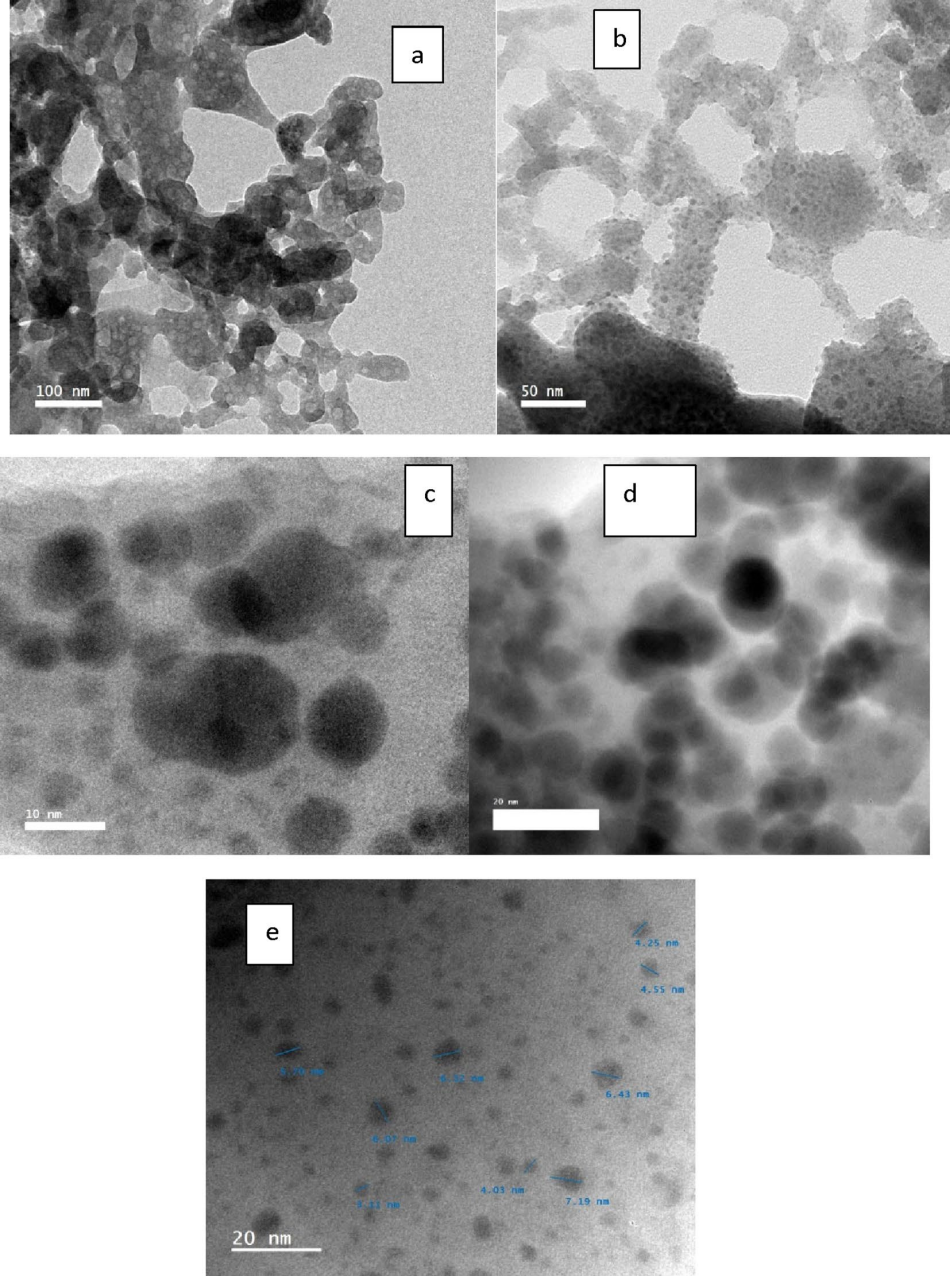
TEM of BS glass samples.

Figure 3 shows the TEM of the current glass samples. The absence of granules is evident in the figure, confirming the amorphous nature of the samples. The TEM image confirms the FTIR’s findings. TEM has examined the surface morphology (Fig. 3a). It is evident that a heterogeneous glassy phase (the black background) contains a large number of solitary spherical particles of various sizes, indicating that the glass structure is entirely amorphous. Large particles (such as stone) appear to form by aggregating smaller ones. As seen in Fig. 3b, these particles are more numerous and larger in Bi_2_O_3_–50B_2_O_3_ glass, forming varying-sized clusters. Presumably, the spherical particles in Fig. 3c, d are primarily composed of amorphous Bi_2_O_3_, while they might also include tiny α-Bi_2_O_3_ phase crystallites. Conversely, the glassy phase, or dark background, is thought to be a borate-rich phase made up of modified BO_4_ and symmetric BO_3_ units. With a diameter of approximately 10 nm, it is evident from Figs. 3c–e that the BS glass samples are nano-shaped.

## ATR-FTIR

**Figure 4:**
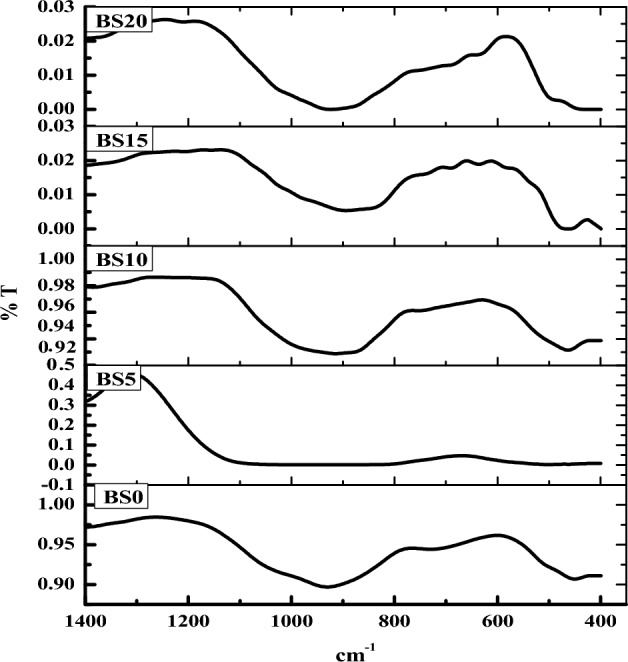
IR spectra of the glass system samples.

The band at about 450 cm^ −1^, which is present in all glasses, is formed by overlapping Bi–O bending vibrations in BiO6 octahedral units and Si–O–Si bending in SiO_4_ tetrahedral units, according to the IR study of the glass system (Fig. 4)^32,33^. As the ZnO concentration rises, the intensity of this band falls due to the reduction in the BiO_6_ group. Bi-O- stretching vibrations, or non-bridging oxygen in BiO_6_ units, may be the source of the absorption band in the 610–623 cm^ −1^ regions. The band at 699–712 cm^ −1^ is thought to be responsible for the bending vibrations of B–O links in the borate network^32,34^. A faint band at 885 cm^ −1^ has been seen as a result of the non-bridging oxygen of BO_4_ groups overlapping with the symmetrical stretching vibrations of the Bi–O bond in the BiO_3_ and Si–O stretching with non-bridging oxygen [4, 11, and 12]. The band overlaps the symmetric stretching vibration of the SiO_4_ and BO_4_ tetrahedral units in the 1006–1028 cm cm^ −1^ regions^32,35^. The band in the range of 1060–1075 cm^ −1^ is caused by the Si–O–Si anti-symmetric stretching vibrations of bridging oxygen in tetrahedral and BO_4_ units^36^. As the zinc level rises, the band at about 1060 cm^ −1^ shifts to a higher wave number of 1075 cm^ −1^. The increasing strength of this band indicates an increase in BO_4_ groups. The band at 1370 cm^ −1^ is caused by the stretching and bending vibrations of BO3 groups^37^. The spectra show that there is an increase in intensity in the band between 800 and 1200 cm^ −1^ and a decrease in strength in the band around 1370 cm^ −1^. This suggests that the inclusion of bismuth modifies the glass network. The spectra were first normalised at 1280 cm^ −1^ and subtracted for the baseline. It is evident from the spectra that the same vibrational modes with substantially varying intensities and bandwidths are centred within the experimental error around the same places in this range. This behaviour could be explained by the overlapping of the infrared modes from all existing nanotube diameters, notably below 1300 cm^ −1^. This interpretation appears to be supported by TEM evidence. In actuality, the TEM observations of a wider range of glass sample diameters in samples reflect a more pronounced broadening of the infrared bands for those samples.

## Radiation Shielding Results

### Linear and Mass Attenuation Coefficients (LACs, MACs)

The experimental values of LAC for BS0, BS1, BS2, BS3 and BS4 at 0.0595 MeV are 0.715, 1.757, 3.072, 4.727 and 6.46 cm^−1^, respectively, as presented in Table 2‘s calculations for nano samples. The LAC values for the BS0, BS1, BS2, BS3 and BS4 glasses are 0.141, 0.181, 0.211, 0.244 and 0.267 cm^−1^ at 1.408 MeV, respectively. Because Bi_2_O_3_ subrogated B_2_O_3_, which had a lower molecular weight (MW = 69.6182 g mol^−1^) and a lower density (ρ = 8.9 g cm^−3^ and MW = 465.96 g mol^−1^), the glass LAC increased as a result of the Bi2O3 modification.

**Table 2:** Linear attenuation coefficients of the nano glass samples.

E (keV)	μ_BS0_ (cm^−1^)	μ _BS1_ (cm^−1^)	μ _BS2_ (cm^−1^)	μ _BS3_ (cm^−1^)	μ _BS4_ (cm^−1^)
59.51	0.7153	1.7567	3.0723	4.7267	6.4595
80.99	0.5466	1.0627	1.6748	2.4254	3.1895
121.78	0.4447	1.1432	2.0184	3.1194	4.2990
244.7	0.3442	0.5328	0.7269	0.9673	1.1889
356.01	0.2963	0.4175	0.5295	0.6602	0.7795
661.65	0.2213	0.2947	0.3525	0.4161	0.4677
778.9	0.2035	0.2693	0.3192	0.3741	0.4166
964.08	0.1823	0.2409	0.2855	0.3288	0.3612
1173.24	0.1608	0.2083	0.2450	0.2830	0.3135
1332.5	0.1503	0.1928	0.2272	0.2616	0.2857
1408.01	0.1411	0.1814	0.2112	0.2438	0.2668

The preponderance of distinct photon interactions in different energy areas is what caused the discrepancies in the MAC values shown in Table 3 for the synthesised glasses with or without metal oxides at different incident gamma ray energies. As indicated in Table 3, the MAC values rose as the glass samples’ bismuth oxide content grew from x = 5% to x = 20%.The range of values is 1.41 MeV to 0.0595 MeV. Therefore, at 0.0595 MeV, the MAC for pure glass BS0 is 0.2815 cm^2^/g for glasses BS1, BS2, BS3, and BS4 samples, the MAC values are 0.5424, 0.8184, 1.104, and 1.3948 cm^2^/g; and at 1.408 MeV, the MAC values for the glasses BS0, BS1, BS2, BS3, and BS4 are 0.0555, 0.0560, 0.0563, 0.0569, and 0.0576 cm^2^/g, respectively. However, according to XCOM calculations, the MAC values at 0.0595 MeV are 0.2323, 0.4408, 0.6493, 0.8578, 1.066 cm^2^/g for BS0, BS1, BS2, BS3, and BS4 samples; and at 1.41 MeV, the MAC values are 0.529, 0.0529, 0.053, 0.053, and 0.0531 for BS0, BS1, BS2, BS3, and BS4 glass. These numbers make it evident that the MAC estimated using the experimental technique was higher than the XCOM findings. Thus, adding Bi_2_O_3_ to the glass improves its ability to shield. However, the photon’s energy determines how quickly the MAC grows. Table 3 shows that the addition of Bi_2_O_3_ results in a rise in MAC at low energy. Now let’s look at Bi_2_O_3_ and see how the MACs are rising for the different stated energies. As a result, the addition of Bi_2_O_3_ at high energies raised the MAC. When the impact of particle size on the MAC values is compared, it is evident that the MAC for BS4 is higher than that of BS0 as well. This is consistent with the Lambert–Beer law, which states that as photon energies increase, the mass attenuation coefficients decrease and LAC values decrease.

**Table 3:** The calculated and theoretical values of MACs (cm^2^/g) for nano and bulk glass samples respectively.

E (keV)	E (keV	BS0 Density (g/cm3) = 2.541 ± 0.082		BS1 Density (g/cm3) = 3.239 ± 0.13		BS2 Density (g/cm3) = 3.754 ± 0.08		BS3 Density (g/cm3) = 4.281 ± 0.02		BS4 Density (g/cm3) = 4.631 ± 0.004	
		EXP	X-COM	EXP	X-COM	EXP	X-COM	EXP	X-COM	EXP	X-COM
59.51	59.51	0.2815	0.2327	0.5424	0.4421	0.8184	0.6514	1.1041	0.8608	1.3948	1.0700
80.99	80.99	0.2151	0.1778	0.3281	0.2668	0.4461	0.3558	0.5666	0.4447	0.6887	0.5337
121.78	121.78	0.1750	0.1451	0.3529	0.2889	0.5377	0.4326	0.7287	0.5763	0.9283	0.7201
244.7	244.7	0.1355	0.1132	0.1645	0.1353	0.1936	0.1573	0.2260	0.1794	0.2567	0.2014
356.01	356.01	0.1166	0.0985	0.1289	0.1064	0.1411	0.1143	0.1542	0.1223	0.1683	0.1302
661.65	661.65	0.0871	0.0761	0.0910	0.0775	0.0939	0.0789	0.0972	0.0802	0.1010	0.0816
778.9	778.9	0.0801	0.0707	0.0832	0.0715	0.0850	0.0723	0.0874	0.0732	0.0899	0.0740
964.08	964.08	0.0718	0.0639	0.0744	0.0643	0.0761	0.0647	0.0768	0.0650	0.0780	0.0654
1173.24	1173.24	0.0633	0.0580	0.0643	0.0581	0.0653	0.0583	0.0661	0.0584	0.0677	0.0586
1332.5	1332.5	0.0591	0.0544	0.0595	0.0544	0.0605	0.0545	0.0611	0.0546	0.0617	0.0546
1408.01	1408.01	0.0555	0.0529	0.0560	0.0529	0.0563	0.0530	0.0569	0.0530	0.0576	0.0531

### Half and tenth value layer, mean free path (HVL, TVL, MFP)

Table 4 lists the examined H%, HVL, TVL, and MFP values for the nano glass samples. The width of the glass material needed to reduce photon intensity to half the incident value is predicted by HVL^26,27^. The material composition and the energy of the incoming photon have an impact on the HVL, demonstrating a positive correlation between the photon energies and the simulated HVL. At 0.0596 MeV, the thinnest HVL was seen, while at high energy (1.408 MeV), the thickest HVL. It is possible to raise the material density and electron density of the sample being tested by substituting Bi_2_O_3_ for B_2_O_3_. As a result, there are more gamma photon collisions, which cause the entering photons to be more attenuated. Table 3 illustrates that the thinnest HVL is found in BS4 glass containing 20% Bi_2_O_3_, whereas the thickest HVL is found in BS0 glass without Bi_2_O_3_. In order to reduce exposure dosage or absorbed dose to a tenth of the value computed without the material, the TVL predicts a specific substance thickness that has attenuated gamma radiation to a range^26,27^. The simulated TVL and photon energies show a positive association, as seen in Table 4. The highest energy (1.408 MeV) produced the thickest TVL, whereas the lowest TVL was seen at (0.0596 MeV). The expected TVL values are significantly impacted by the material composition as well. The mean distance between two beams in a gamma-ray collision is predicted using MFP^13,22^. Similar trends and arguments can be seen in the estimation of MFP as well as in the HVL section (see Table 3). The BS0 glass sample had the maximum MFP, whereas the BS4 glass sample had the lowest MFP.

**Table 4:** The values of HVL, TVL and MFP.

E (keV)	BS0 H % = 22.5066			BS1 H % = 28.6891			BS2 H % = 33.2507			BS3 H % = 37.9185			BS4 H % = 41.0186		
	MFP (cm)	TVL (cm)	HVL (cm)	MFP (cm)	TVL (cm)	HVL (cm)	MFP (cm)	TVL (cm)	HVL (cm)	MFP (cm)	TVL (cm)	HVL (cm)	MFP (cm)	TVL (cm)	HVL (cm)
59.51	1.398	3.219	0.969	0.569	1.311	0.395	0.325	0.749	59.51	0.212	0.487	0.147	0.155	0.356	0.107
80.99	1.830	4.213	1.268	0.941	2.167	0.652	0.597	1.375	80.99	0.412	0.949	0.286	0.314	0.722	0.217
121.78	2.249	5.178	1.559	0.875	2.014	0.606	0.495	1.141	121.78	0.321	0.738	0.222	0.233	0.536	0.161
244.7	2.905	6.689	2.014	1.877	4.322	1.301	1.376	3.168	244.7	1.034	2.380	0.717	0.841	1.937	0.583
356.01	3.375	7.770	2.339	2.395	5.516	1.660	1.889	4.348	356.01	1.515	3.488	1.050	1.283	2.954	0.889
661.65	4.518	10.404	3.132	3.393	7.812	2.352	2.837	6.532	661.65	2.403	5.534	1.666	2.138	4.923	1.482
778.9	4.914	11.316	3.406	3.713	8.549	2.573	3.132	7.213	778.9	2.673	6.155	1.853	2.401	5.528	1.664
964.08	5.484	12.627	3.801	4.151	9.557	2.877	3.503	8.065	964.08	3.041	7.002	2.108	2.769	6.375	1.919
1173.24	6.218	14.318	4.310	4.802	11.057	3.328	4.082	9.399	1173.24	3.534	8.136	2.449	3.190	7.344	2.211
1332.5	6.654	15.321	4.612	5.186	11.941	3.595	4.402	10.136	1332.5	3.823	8.802	2.650	3.500	8.059	2.426
1408.01	7.086	16.316	4.912	5.512	12.692	3.821	4.735	10.903	1408.01	4.103	9.446	2.844	3.748	8.630	2.598

### Xeff of nano bismuth boro-silicate glass comparing with lead glass

**Figure 5:**
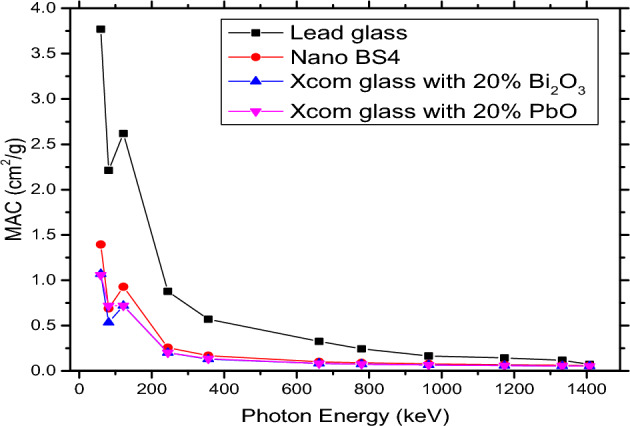
Comparison between MAC for glass with PbO, Bi2O3, BS4 and Lead glass.

When the results of MAC Nano glass samples 20% BS4 are compared to the theoretical results of MAC through XCOM of lead oxide 20% glass, as Fig. 5 illustrates, the nano samples are significantly better. However, when bismuth oxide 20% XCOM samples are compared, we find that they are comparable to XCOM lead oxide 20%. When the glass samples (BS4) used in this study are compared to lead glass used in radiation protection in the medical field, and its density is (4.46 g/cm^3^) and heaviness (39.5% For the Bi and Pb (90.534 keV and 88.005 keV, respectively), we observed a reduction following the k-edge that edges had non-continuous peaks in this energy range^16,38^. Table 5 tabulates the values of BS4 thickness with respect to 1 mm lead glass.

**Table 5:** The Effective BS4 thickness values equivalent to1mm Glass Lead in (mm).

E (keV)	BS4 H % = 41.0186		Glass Lead H % = 39.5		Effective BS4 thickness equivalent to 1 mm Glass Lead in (mm)
	μ_BS4_ (Cm^−1^)	HVL (Cm)	μ_Glass lead_ (Cm^−1^)	HVL (Cm)	
59.51	6.4595	0.107	16.8113	0.041	2.60
80.99	3.1895	0.217	9.865	0.070	3.09
121.78	4.2990	0.161	11.6793	0.059	2.71
244.7	1.1889	0.583	3.9112	0.177	3.29
356.01	0.7795	0.889	2.5400	0.273	3.26
661.65	0.4677	1.482	1.4499	0.478	3.10
778.9	0.4166	1.664	1.0863	0.638	2.61
964.08	0.3612	1.919	0.7343	0.944	2.03
1173.24	0.3135	2.211	0.6389	1.085	2.04
1332.5	0.2857	2.426	0.5192	1.335	1.82
1408.01	0.2668	2.598	0.3229	2.146	1.*21*

### The transmission factor (TF%)

**Figure 6:**
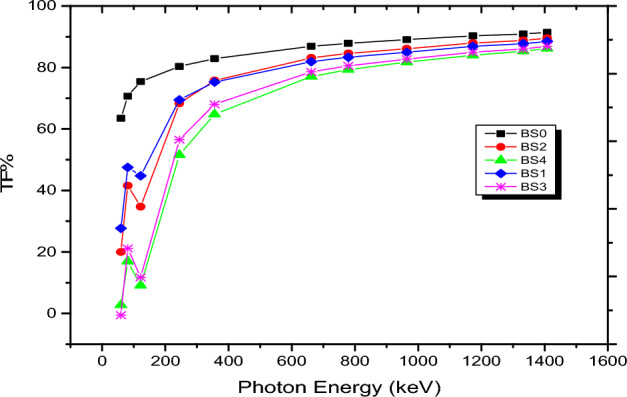
Shows TF% measurements for the investigated BS glass samples.

A crucial metric for estimating how many photons will enter the sample under study is TF%. Figure 6 displays the TF variation with incident photon energy for all glass samples, BS0 through BS4.The primary TF parameter is the incident photon energy, and the TF value rises linearly with the photon energy. The expected TF is smaller at low energies (0.0596 MeV), falling between 63.494 and 2.738% for glass BS0 and BS4, respectively. Conversely, the maximum TF is obtained at higher gamma photon energies (1.408 MeV), where the TF varies between 91.429 and 86.191% for glass BS0 and BS4. The photon penetration power (PP) and the decrease in photon wavelength (λ) lead to a direct proportion between the TF and the incoming energy (PP ∧ E λ).

### The radiation protection efficiency (RPE%)

**Figure 7:**
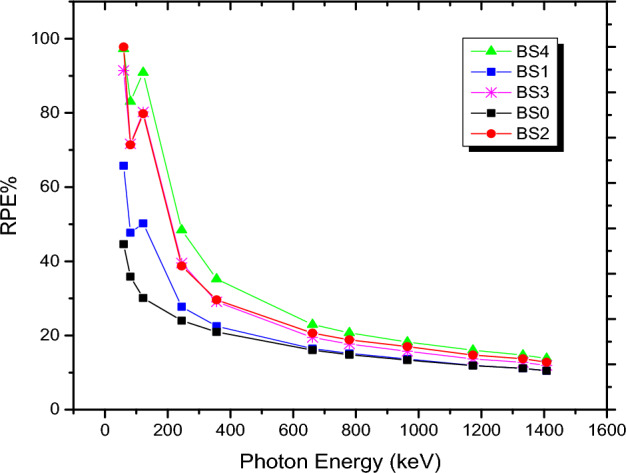
RPE% measurements for the investigated BS glass samples.

Figure 7 shows the RPE% variations for all BS0–BS4 glass samples with the incoming photon energies. As photon energy increases, RPE values fall. The glasses BS0 and BS4 were expected to have the lowest RPE at low energies (0.0596 MeV), with a decrease in range of 36.51% to 97.26%. Otherwise, glasses BS0 and BS4 had the lowest RPE, which varied between 8.57 and 13.81 percent, at higher gamma photon energy (1.408 MeV). It is clear that glasses containing 20% Bi_2_O_3_ have a good shielding ability at 0.059 MeV, which indicates that nearly all photons at this energy can be stopped by the glass.

### Equivalent atomic number (Zeq) and buildup factors (EBF and ABF)

**Figure 8:**
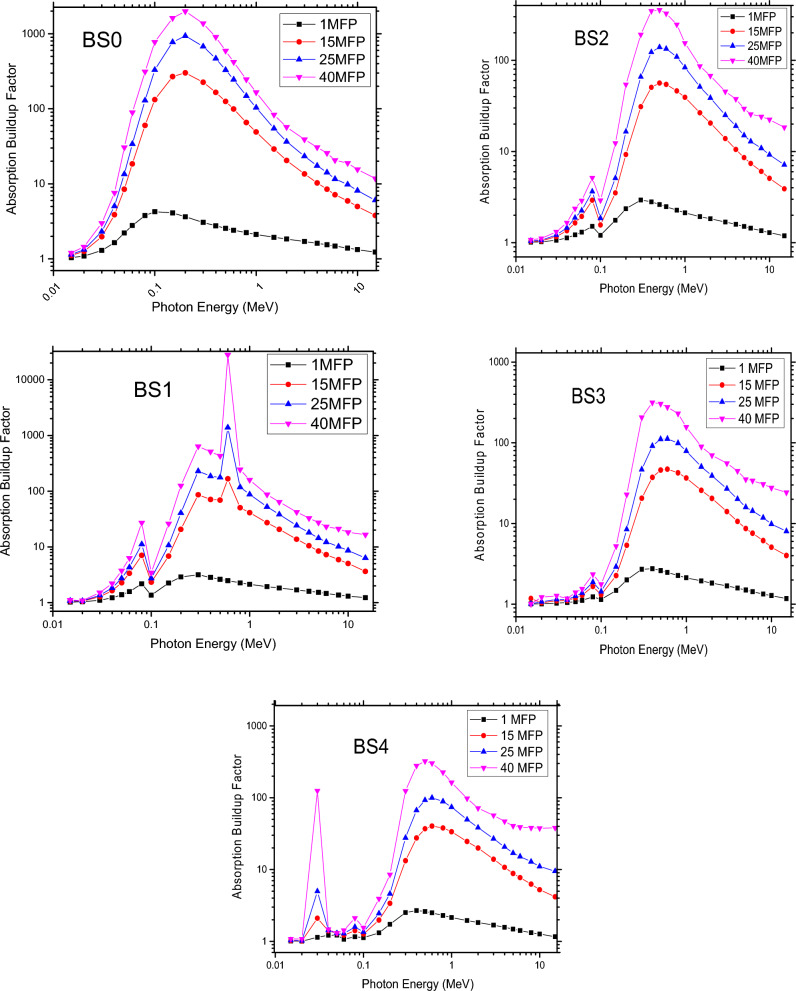
The Absorption Buildup Factor at 1, 15, 25 and 40 MFP for glass samples.

**Figure 9:**
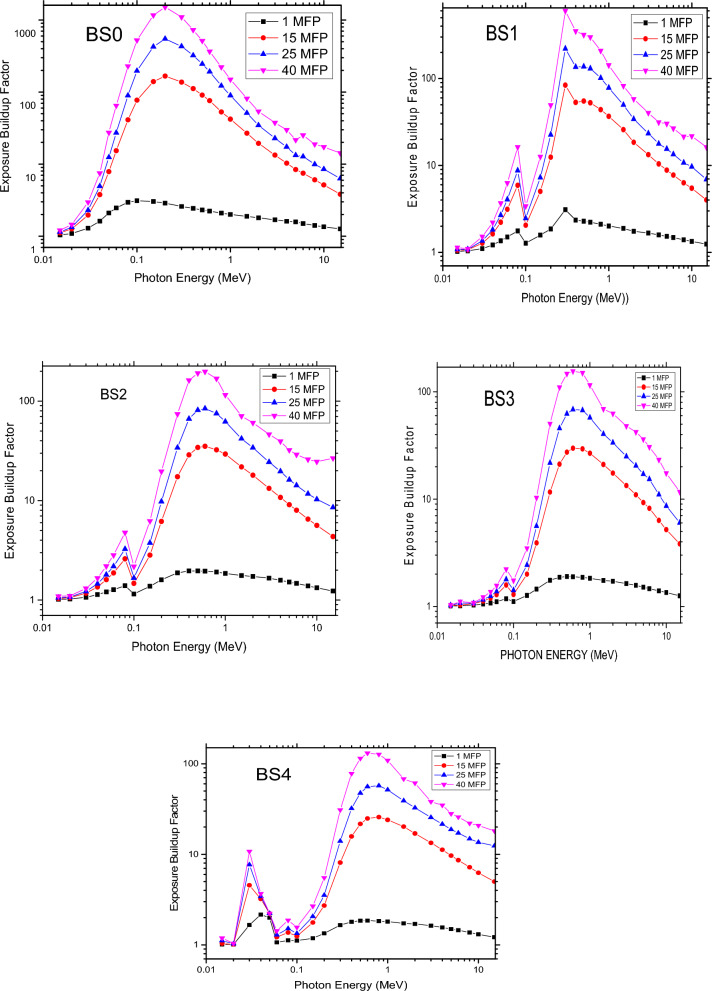
The Exposure Buildup Factor at 1, 15, 25 and 40 MFP for glass samples.

In shielding calculations, the Buildup factors are modification variables that take the effects of extra stray radiation in the medium into consideration^16,39^. The equation of equivalent atomic number (Zeq) was used to characterise them. The interaction of gamma rays is the basis for the Zeq of the glasses. The following formula is used to determine the glass’s Zeq. Photoelectric and electron pair-generating interactions are responsible for the lowest Zeq values seen in the low- and high-energy ranges, while Compton’s scattering effect is responsible for the highest Zeq values found in the middle-energy region. Moreover, Zeq rises when the percentage of bismuth condensation in glass increases. In the energy range of 0.015 to 15 MeV, the accumulation factors (ABF and EBF) as a function of gamma ray energy were also computed from depths of 1, 15, 25, and 40 mean free pathways (See Figs. 8 and 9). Because there are more scattered photons when the Mean Free Path (MFP) increases, it has been observed that the Absorption Buildup Factor (ABF) and Exposure Buildup Factor (EBF) also rise^16,39^. Photons stay in the material longer because they are scattered with energy loss as opposed to being absorbed. Due to photoelectric interactions, ABF and EBF for all glasses have the lowest values related to low gamma rays in this energy range, whereas paired interactions yield the maximum values, which lead to total absorption of photon energies at highlights. Additionally, because of Compton scattering, photon energy is lowered but not entirely absorbed, bringing the energy at the medium very close to a maximum. It was determined that when Bi2O3 was added, the ABF and EBF values decreased at low and medium energies and increased at high energies (> 1 MeV). The partial absorption of photon energy by the glass samples’ ABF and EBF in this work leads to an energy drop and a shift in photon direction. Thus, the photons in the medium as accumulation factors are related to these results of the numerous scattering processes. Because of this, adding Bi2O3 to the glass lowers the photon accumulation factor, which means that instead of being absorbed, the photons are dispersed as a result of energy loss, demonstrating that the photons remain in the sample for a longer period of time^16,39^.

## Conclusion

This study evaluated Bismuth Boro-Silicate glasses as a potential substitute for Lead glass in gamma-ray shielding applications. The environmentally beneficial Bismuth Boro-Silicate glasses were investigated for their gamma ray shielding capabilities. Experimental and theoretical studies of the shielding parameters were conducted under gamma rays with energies of 59.51, 80.99, 121.78, 244.7, 356.01, 661.65, 778.9, 964.08, 1173.24, 1332.5, and 1408.01 keV. The network structure of the Boro-Silicate glass samples is modified by the addition of Bi3 + , which has a high polarizability. When our glass samples were compared to shielding materials currently in use in industrial applications, the lower HVL and MFP values found for our glass samples for the majority of the composition range proved the superior performance of our glass samples.

## Data Availability

The data set used for this analysis cannot be fully shared publicly because of restrictions relating to the copyright ethics for authors in our nuclear lab which conducted our research experiments. The datasets generated and analyzed in this study are available from the corresponding author on reasonable request.
